# Leiomyosarcoma of the inferior vena cava

**DOI:** 10.1016/j.radcr.2023.09.069

**Published:** 2023-11-10

**Authors:** Maria Antonietta Di Pilla, Marco Alex Capuano, Mariangela Rossi, Gianni Di Pilla, Rocco Minelli, Paolo Pizzicato, Antonio Rossi, Giuseppe Paviglianiti, Donatella Irace, Gianfranco Vallone, Antonio A.H. Salvia, Maria Cristina Smaldone, Valentina Cariello, Raffaele Zeccolini, Eugenio Rossi

**Affiliations:** a Life and Health Department “V. Tiberio”, University of Molise, Campobasso, Italy; b Department of Radiodiagnostics “F. Veneziale”, Molise Regional HealthCompany (ASREM), Isernia, Italy; c U.O.S.D. Diagnostic Imaging P.O. Pausilipon - AORN Santobono –Pausilipon, Naples, Italy; d Campus Biomedico University, Rome, Italy; e U.O.C. Pediatric Radiology PO G. Di Cristina-ARNAS Civico Benfratelli, Palermo, Italy; f A.O.U. Federico II, Department of Translational Medical Sciences, Pediatric Section, Naples, Italy; g Department of Precision Medicine, University of Campania “Luigi Vanvitelli”, Naples, Italy

**Keywords:** Leiomyosarcoma, Inferior vena cava, Retroperitoneal masses, Integrated imaging

## Abstract

Inferior vena cava leiomyosarcoma is a rare malignant mesenchymal tumor that originates from the smooth muscle cells of the venous media and is more frequent in females in the V-VI decade of life. Due to scarce and specific symptoms, diagnosis is not simple, and often metastases to the liver, lungs, and/or lymph nodes are already present. A 44-year-old male patient arrives at our institution presenting diffuse abdominal pain and a sense of weight associated with lumbar pain. He showed nothing relevant except for a moderate consumption of alcohol. Ultrasound examination of the abdomen revealed liver enlargement with hyperechoic nodularity and clear margins. Furthermore, the presence of a voluminous solid nodular formation was found, with an inhomogeneous echostructure and moderate vascularization on Doppler. Inferior vena cava leiomyosarcoma is a rare malignancy. The diagnosis is usually established after surgery, however, recurrences are common and the role of chemoradiation therapy remains to be defined. The only potential treatment is surgical resection with possible vessel reconstruction and en bloc removal of adjacent structures with 5 and 10-year survival rates of 49% and 29%, respectively.

## Introduction

Leiomyosarcoma of the inferior vena cava (VCI) is a very rare mesenchymal tumor associated with an extremely poor prognosis. It accounts for less than 1 in 100,000 of all malignancies and about 0.5% of adult soft tissue sarcomas. Fewer than 450 cases are reported in the literature [1], the first was described by Pearl during an autopsy in 1871 [2]; it is, nonetheless the most common primary tumor of IVC. IVC leiomyosarcomas occur predominantly in females with a female: male ratio of approximately 3:1. These tumors tend to be more common in the 5th-6th decade of life [1].

These lesions are classified according to the segment involved: inferior (infrarenal, 34%), medium (from the renal vein to the hepatic veins, 42%), and superior (from the hepatic veins to the right atrium, 24%) [3]; they are also associated with 3 tumor growth patterns: extraluminal (62%), intraluminal (5%) and combined (33%). Intraluminal and mid-tract ones have a better prognosis as they are usually discovered more prematurely. Patients may be asymptomatic or present nonspecific symptoms such as malaise, weight loss, nausea, vomiting, and abdominal pain [1]. More specifically, patients with lesions of the upper portion of the IVC often experience nausea, weight loss, Budd-Chiari syndrome, and lower limb edema, those with involvement of the middle segment experience pain in the epigastrium and right hypochondrium, and finally, those with infrarenal lesions show pain in the right iliac fossa and back with edema of the lower limbs [3]. Tumor fragments can detach and embolize in the pulmonary arteries or even in the right atrium causing pulmonary emboli, tricuspid valve impairment, and cardiac arrhythmia [1]. At the macroscopic level, leiomyosarcomas are generally well-circumscribed fleshy masses that can show hemorrhages, necrosis, or cystic modifications; the extraluminal ones often reach large dimensions (> 10 cm) in the retroperitoneal space before giving symptoms; on the contrary, the intraluminal ones are small and firmly attached to the vessel wall and due to their smaller size they do not undergo hemorrhage or necrosis. Microscopically these foams show atypical patterns of spindle cells with elongated nuclei and positivity for smooth muscle actin, caldesmin, desmin, and sometimes also for vimentin and epithelial membrane antigens. Interestingly, high expression of cytoplasmic β-catenin is associated with higher distant recurrence and lower survival. Furthermore, in many vascular leiomyosarcomas, the mutation of the Pten gene is also found, causing dysregulation of phosphatidylinositol 3-kinase (PI3K), hyperactivation of Akt, increased cell proliferation, inhibition of apoptosis and defective double-strand break repair [1].

## Case presentation

**Fig. 1:**
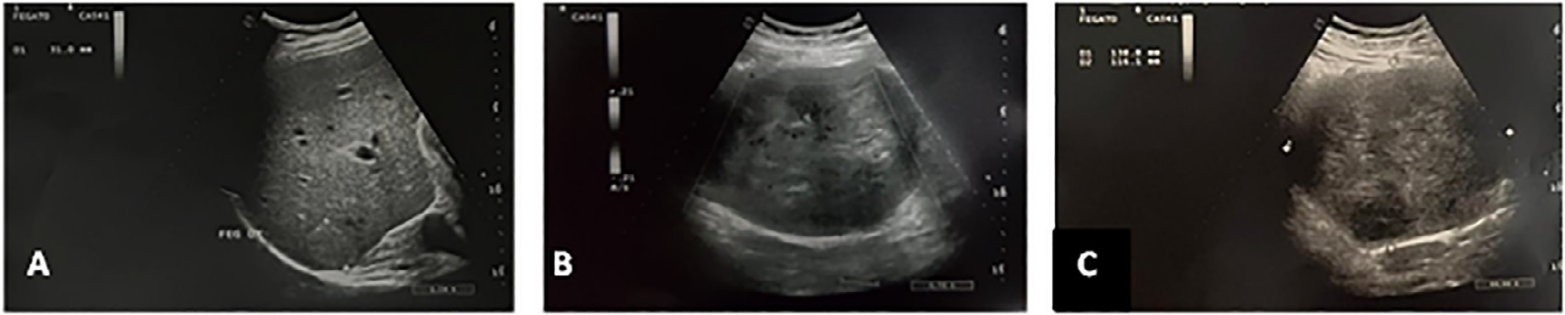
Angioma of the VI-VIIth segment: a nodukar formation of 3 mm, hyperechoic and with clear margins (A). Voluminous solid nodular formation of an expansive character measuring 14 × 11.6 cm whose echoctructure was heterogenous (C), and with a significant vascularization at Doppler (B).

**Fig. 2:**
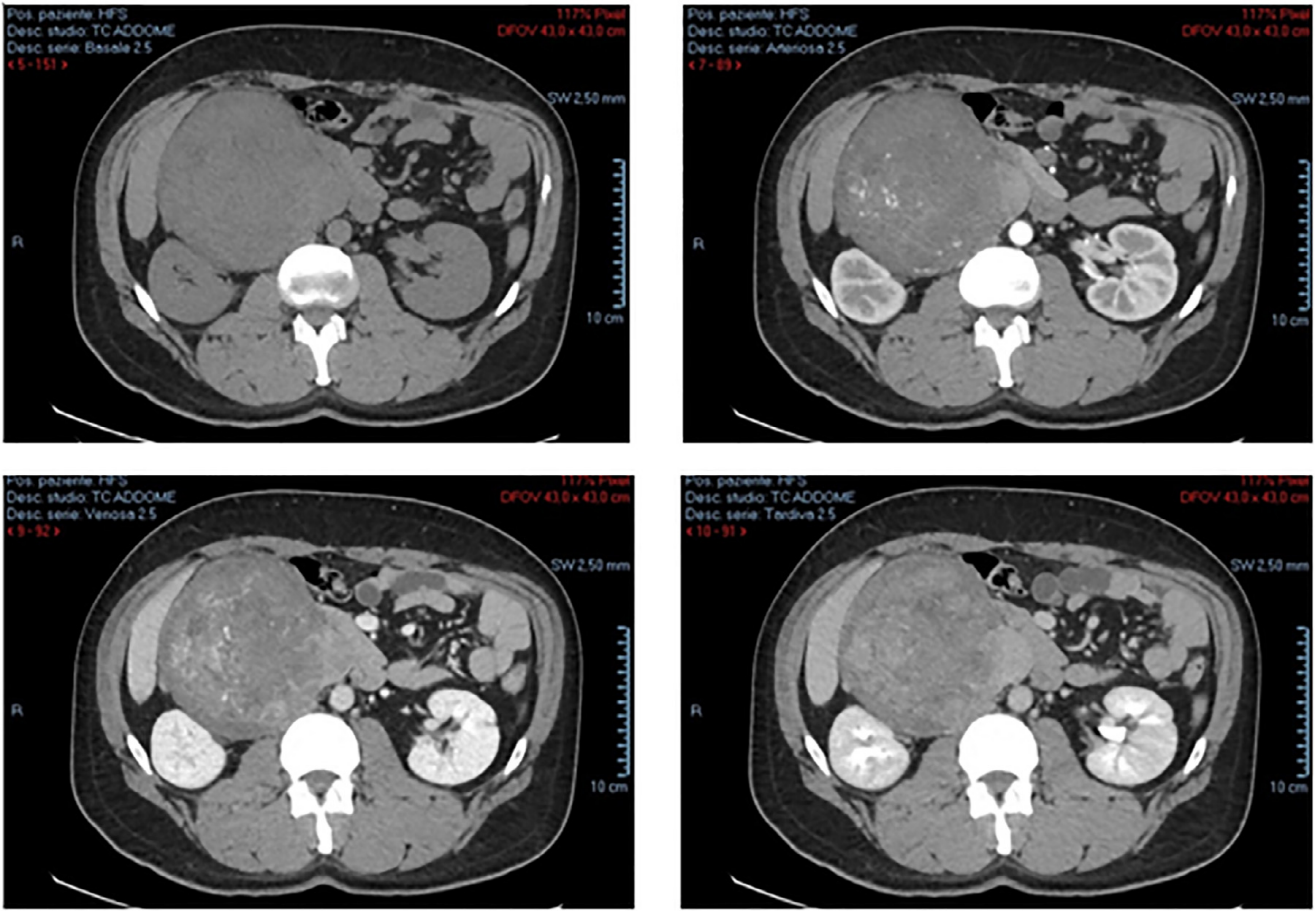
Voluminous right retroperitoneal solid expansive process, that was unevenly isodense with respect to the kidney at the basal scan and then presented a predominantly centronodular enhancement and with an embryonic hair-shaped appearance in the arterial scan, which in turn became more dense in the portal scan and even more at equilibrium.

**Fig. 3:**
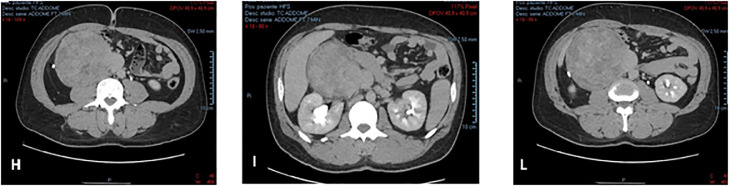
Expansive formation in close contiguity with the VIth hepatic segment anterolaterally (I), which displaces posterolaterally the right urether determining a moderate extrinsic compression on the right kidney, characterized by mild hydro-nephrosis (H, I). IVC with semicircular morphology and clear angles (I, L).

We present the case of a leiomyosarcoma of the VCI found in a 44-year-old male patient who came to our observation to perform an abdominal ultrasound following repeated widespread abdominal pain and a sense of weight associated with lumbar pain; in his anamnesis, he does not show anything relevant except for a discreet consumption of beer. On ultrasound examination, despite marked intestinal meteorism, it was possible to detect: an increase in the volume of the liver with a nodularity of 3 cm at the border between VI and VII segment, hyperechoic in structure and with clear margins, compatible with an angioma; beyond a substantial negativity of the remaining abdominal organs we found in the subhepatic area, a conspiucous solid nodular formation, of an expansive nature, whose maximum dimensions were 14 × 11.6 cm and which appeared echostructurally inhomogeneous with an iso-hypo-hyperechoic appearance and a discrete vascularization on color Doppler (Fig. 1). This finding suggested the opportunity of completing the diagnosis by CT examination of the abdomen with contrast medium, also extended to the chest and skull. CT examination was performed before and after intravenous iodized contrast medium infusion (Iomeron 400, 110 cc) with a multiphasic spiral technique. The latter confirmed the presence of a voluminous right retroperitoneal solid expansive process which was unevenly isodense with respect to the kidney in the basal scan to then show a predominantly centronodular impregnation with a capillariform imbricated appearance in the arterial phase, which in turn became thicker in the portal phase and even more at 3 minutes after the contrast medium infusion (Fig. 2). This mass showed close contiguity with the sixth hepatic segment anterolaterally and displaced the urether posterolaterally to the right, causing a moderate extrinsic compression on the ipsilateral kidney which presented moderate hydronephrosis. The salient element though, which oriented our diagnostic hypothesis was the presence of clear angles of obtuse junctions with the ICV which at this level showed a semi-lunar morphology (Fig. 3). The above allowed us to formulate our diagnosis of a lesion originating from the ICV which, due to the aforementioned densitometric characteristics, could be compatible with a rare case of leiomyosarcoma of the vein itself. At the same time, the CT examination confirmed that the hepatic formation detected by ultrasound was to be interpreted as an angioma in relation to the classic globular and centripetal impregnation of the lesion with a tendency to homogenization in the equilibrium phase and homogeneous hyperdensity in the acquisition carried out at 7 minutes; nothing relevant pertaining the remaining abdominal organs as well as to the thorax and skull was found. Following the aforementioned presumed diagnosis, the patient went to another hospital in order to perform a needle biopsy from which leiomyomatous neoplastic proliferation emerged, though it must be noted that in spite of the smallness of the tissue examined compared to the overall size of the mass it was not possible to exclude the possibility that the latter could have different patterns of aggressiveness in different portions. The patient underwent surgery for the removal of the caval neoformation and reconstruction by means of the peritoneum of the ICV; the histological examination therefore revealed a smooth muscle spindle cell mesenchymal proliferation whose morphological and immunohistochemical findings are compatible with the diagnosis of G2 caval leiomyosarcoma.

**Fig. 4:**
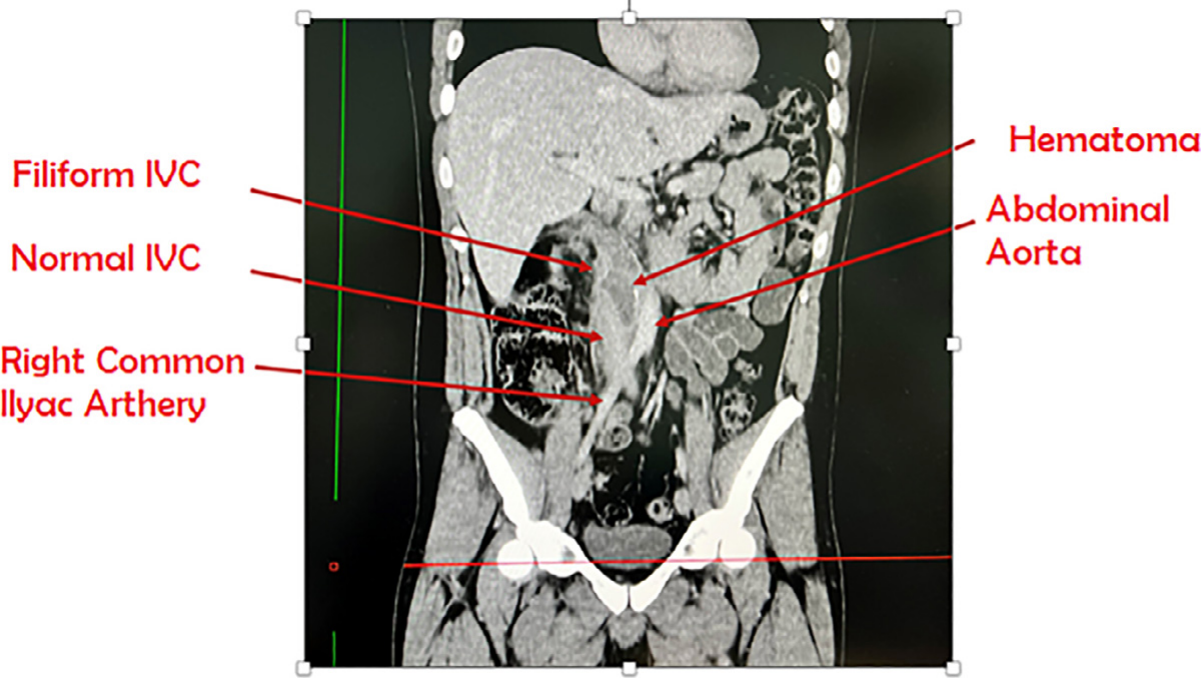
Clear reduction of the postsurgical hematoma, along the side of segment IVC (9 cm), from the iliac bifurcation (where the ICV assumes a filiform appearance) to the confluence of the renal veins upstream of which the configuration if the cava is normal.

During hospitalization the patient underwent 2 transfusions; empiric antibiotic therapy + urine-blood culture and a chest X-ray for the onset of fever; and anticoagulant therapy; Abdominal CT with and without administration of contrast medium, the latter showed the presence of a fluid-superfluid collection with predominantly blood density at the level of the IVC (para-subrenal) with a cranio-caudal extension of 13.4 cm of which we have no images. The patient was discharged with anticoagulant therapy performed with Fondaparinux for 6 months and an abdominal CT with and without administration of contrast medium was prescribed to be performed at our hospital. The latter showed a clear reduction of the postsurgical hematoma along the anterior surface of the IVC, however the aforementioned collection has an extension of 9 cm that goes from the iliac bifurcation (where the IVC assumes a threadlike appearance) up to the confluence of the veins upstream of which the cava configuration is normal (Fig. 4). Finally, to date, we know that the patient is following a chemotherapy regimen based on epirubicin, ifosfamide, and MESNA.

## Discussion and conclusion

The leiomyosarcoma of the VCI is a rare mesenchymal tumor that originates from the smooth muscle cells of the venous media, represents 2% of all leiomyosarcomas, it is more frequent in females in the V-VI decade of life and at the time of diagnosis in 35%-50% of cases there are metastases to the liver, lung and/or lymph nodes; it can affect the lower, middle (as in the case described) or upper venous tract and, based on growth, can be extraluminal (as in the case described), intraluminal or combined. It is often asymptomatic as it has slow growth or may present nonspecific symptoms such as pain, Budd-Chiari syndrome if infrarenal, nephrotic syndrome if of the middle segment, and lower limb edema if of the lower tract, intraluminal and middle segment have a better prognosis for early diagnosis. Ultrasound is often the first-level examination, but derived findings are usually nonspecific since the echo structural heterogeneity can also evoke masses of a different nature such as retroperitoneal sarcomas; moreover, since these are often voluminous masses, it is difficult to topographically locate the form in a precise manner. More particularly in ultrasound, the tumor is heterogeneously hypoechoic; color Doppler may show absent or abnormal IVC flow when an intraluminal form is detected, however, discrete intratumoral vascularization may also be present. When the tumor is exclusively extraluminal, its solid components are generally isoechoic with respect to the liver and may contain internal cystic spaces [1]. Unfortunately, limitations include operator dependency and artifacts in meteoric patients. Computed tomography with contrast medium. therefore represents the gold standard [4] as with its excellent spatial resolution, as well as ready availability, it quite clearly defines the contiguous structures, as well as the intraluminal involvement of the vessel. More specifically, the arterial phase contrastography can allow a better evaluation of the relationship between the tumor and the main adjacent arterial structures for preoperative planning and in order to identify hypervascular metastases; the venous phase instead shows a better opacification of the IVC. Sagittal and coronal reconstructions also help identify the cranio-caudal extent of vessel involvement. A particularly useful imaging feature is an imperceptible IVC at the point of maximum contact with the mass clarifying its origin as vascular rather than retroperitoneal [5], conversely a mass that compresses the IVC suggests its origin outside the vessel; finally, extraluminal development usually follows tissue planes of least resistance and displaces adjacent organs [1]. CT with contrast medium is also a useful tool for the follow-up of IVC leiomyosarcomas [3]. Liver, lungs, and lymph nodes are common areas of metastases in advanced stages; IVC leiomyosarcoma tends to recur more locally than systemically (16% vs 37%) [1]. The differential diagnosis is mainly with renal carcinomas which in 4%-10% of cases infiltrate the IVC; adrenal cortical carcinomas which infiltrate the IVC in 30% of cases and sometimes show calcifications absent in vascular leiomyosarcoma; primary retroperitoneal neoplasms (primarily leiomyosarcomas and retroperitoneal leiomyomas), but imaging features such as an imperceptible VCI help delineate their site of origin [1]. To date, the only potentially curative treatment is surgical resection with eventual vessel reconstruction by means of the peritoneum with 5- and 10-year survival rates of 49% and 29% respectively; en bloc resection of adjacent structures such as right kidney, adrenal gland, and/or gallbladder may be required. Postoperative complications occur in 18%-30% of patients and commonly include lower extremity edema and kidney failure; infrarenal leiomyosarcoma is associated with a lower rate of postsurgical complications than that of the upper segment [1,8]. Histological grade is the most important prognostic factor for adult soft tissue sarcomas and is in particular the best predictor of the likelihood of distant metastases and patient survival [3,6]. The neoadjuvant-adjuvant role of chemotherapy (combination of decarzabine, doxorubicin, cyclophosphamide, and ifosfamide /cisplatin) and radiotherapy is still incompletely understood, however, both therapeutic strategies may be useful before resection in unresectable borderline tumors (such as those extending circumferentially to 180° around the abdominal aorta or superior mesenteric artery) to reduce tumor size and increase resectability rate [1,7].

## Patient consent

With this item we state that informed consent was acquired from our patient for inclusion in the associated study.
